# No evidence for altered intracellular calcium-handling in airway smooth muscle cells from human subjects with asthma

**DOI:** 10.1186/s12890-015-0009-z

**Published:** 2015-02-13

**Authors:** David Sweeney, Fay Hollins, Edith Gomez, Rajendra Mistry, Ruth Saunders, Robert Alfred John Challiss, Christopher Edward Brightling

**Affiliations:** Department of Infection, Immunity & Inflammation, and Institute for Lung Health, University of Leicester, Glenfield Hospital, Leicester, LE3 9QP UK; Department of Cell Physiology & Pharmacology, University of Leicester, Henry Wellcome Building, Lancaster Road, Leicester, LE1 9HN UK

**Keywords:** Asthma, Airway smooth muscle, Calcium, Sarco/endoplasmic reticulum Ca^2+^ ATPase type 2 (SERCA2), Inositol 1,4,5-trisphosphate

## Abstract

**Background:**

Asthma is characterized by airway hyper-responsiveness and variable airflow obstruction, in part as a consequence of hyper-contractile airway smooth muscle, which persists in primary cell culture. One potential mechanism for this hyper-contractility is abnormal intracellular Ca^2+^ handling.

**Methods:**

We sought to compare intracellular Ca^2+^ handling in airway smooth muscle cells from subjects with asthma compared to non-asthmatic controls by measuring: i) bradykinin-stimulated changes in inositol 1,4,5-trisphosphate (IP_3_) accumulation and intracellular Ca^2+^ concentration, ii) sarco/endoplasmic reticulum Ca^2+^-ATPase (SERCA) expression, iii) mechanisms of cytoplasmic Ca^2+^ clearance assessed following instantaneous flash photolytic release of Ca^2+^ into the cytoplasm.

**Results:**

We found no differences in airway smooth muscle cell basal intracellular Ca^2+^ concentrations, bradykinin-stimulated IP_3_ accumulation or intracellular Ca^2+^ responses. Quantification of SERCA2 mRNA or protein expression levels revealed no differences in ASM cells obtained from subjects with asthma compared to non-asthmatic controls. We did not identify differences in intracellular calcium kinetics assessed by flash photolysis and calcium uncaging independent of agonist-activation with or without SERCA inhibition. However, we did observe some correlations in subjects with asthma between lung function and the different cellular measurements of intracellular Ca^2+^ handling, with poorer lung function related to increased rate of recovery following flash photolytic elevation of cytoplasmic Ca^2+^ concentration.

**Conclusions:**

Taken together, the experimental results reported in this study do not demonstrate major fundamental differences in Ca^2+^ handling between airway smooth muscle cells from non-asthmatic and asthmatic subjects. Therefore, increased contraction of airway smooth muscle cells derived from asthmatic subjects cannot be fully explained by altered Ca^2+^ homeostasis.

## Background

Asthma, a chronic inflammatory disease, remains a major health care burden affecting over 300 million people worldwide. There is a subset of patients that do not respond adequately to traditional treatments, highlighting an urgent need for new therapies [1,2]. Asthma is characterized by variable airflow obstruction and airway hyper-responsiveness as a consequence of increased airway smooth muscle (ASM) contractility [3,4]. An increasing body of evidence supports the view that ASM is fundamentally altered in asthma compared to non-asthmatic controls, suggesting that abnormalities in these structural cells may also play a critical role in the development of the abnormal physiology in asthma and may contribute to the persistent airway inflammation [5-8]. Critically, there is emerging evidence that ASM from asthmatics is hyper-contractile, as demonstrated by an increased velocity of contraction in response to electrical field stimulation at the single cell level [9] and in cell populations using gel contraction assays [5,10].

Cytosolic calcium concentration ([Ca^2+^]_i_) plays a cardinal role in ASM contraction. An increase in intracellular ASM [Ca^2+^]_i_ is likely to originate from the sarcoplasmic reticulum of the cell, with evidence of a lesser reliance on Ca^2+^-influx across the sarcolemma through voltage-dependent channels than other muscle types [11-14]. Evidence has accrued to show that ASM calcium homeostasis is abnormal in asthma, suggesting abnormal [Ca^2+^]_i_ handling, signalling or storage, as possible underlying mechanisms for this hyper-contractility [11,15,16]. Resolution of an increase in [Ca^2+^]_i_ involves the movement of Ca^2+^ out of the cell through the sarcolemmal Ca^2+^-ATPase or Na^+^/Ca^2+^-exchangers, or into the sarcoplasmic reticulum through the sarco/endoplasmic reticulum Ca^2+^-ATPase (SERCA). SERCA2 is the dominant SERCA isoform expressed in ASM, predominantly as the SERCA2B splice variant, and reduced SERCA2 in ASM from subjects with asthma has been reported and proposed to prolong the time for [Ca^2+^]_i_ to return to baseline following stimulation [11]. To date abnormalities in [Ca^2+^]_i_ handling in ASM from asthmatics have been explored following agonist-induced activation. Such receptor-dependent changes in [Ca^2+^]_i_ are often complex, involving complex signal transduction mechanisms that can modulate release and re-uptake of [Ca^2+^]_i_ at multiple loci. Flash photolysis allows a ‘caged’ form of calcium to be released in a controlled, time-resolved manner to investigate [Ca^2+^]_i_ recovery independently of a need for agonist addition [17]. Furthermore by pharmacological inhibition of SERCA activity it is possible to assess the rates of movement of Ca^2+^ across the sarcoplasmic reticular and/or sarcolemmal membranes to determine if potential asthma-related abnormalities in [Ca^2+^]_i_ handling are observed independently of the need to perturb Ca^2+^ homeostasis via receptor-mediated mechanisms.

We hypothesized that Ca^2+^ handling is abnormal in ASM from subjects with asthma. To test our hypothesis we compared in primary ASM cells from subjects with asthma and non-asthmatic controls: (i) bradykinin-induced changes in intracellular calcium [Ca^2+^]_i_; (ii) SERCA expression, (iii) agonist-stimulated changes in inositol 1,4,5-trisphosphate (IP_3_) accumulation, and (iv) [Ca^2+^]_i_ kinetics assessed following release of a Ca^2+^ load into the cytoplasm by flash photolysis and uncaging.

## Methods

### Subjects and cells

Asthmatic subjects (n = 20) and non-asthmatic controls (n = 18) were recruited from Leicester, UK. Asthma severity was defined by Global Initiative for Asthma (GINA) treatment steps (mild-moderate GINA 1–3, severe GINA 4–5) [4]. Primary ASM cells were isolated from bronchial biopsies and used at passages 2–5. The study was approved by the Leicestershire Ethics Committee and patients gave their written informed consent.

### Intracellular Ca^2+^ imaging

To measure changes in [Ca^2+^]_i_, ASM cells plated at sub-confluence on coverslips were loaded with 2 μM Fura-2 AM (Molecular Probes/Thermo Fisher Scientific) in the presence of 2.5 mM probenecid (Sigma-Aldrich) and 0.04% w/v pluronic F127 (Molecular Probes/Thermo Fisher Scientific), and visualized using an inverted epifluorescence microscope (Nikon Diaphot 200). Changes in Fura-2 fluorescence (F) intensity were measured as a ratio, R, where R = F_340 nm_/F_380 nm_, such that R increases as [Ca^2+^]_i_ increases [18]. Bradykinin (1 μM) was added to ASM cells via perfusion (at 5 mL min^−1^) in physiological saline solution (118.4 mM NaCl, 4.7 mM KCl, 2.0 mM CaCl_2_, 1.2 mM MgCl_2_, 11.1 mM glucose, 10 mM HEPES, pH 7.4) at 37°C.

### Real-time reverse transcription-polymerase chain reaction

Real-time reverse transcription-polymerase chain reaction was performed (SuperScript Vilo cDNA synthesis kit, Express SYBR-GreenER qPCR Supermix Universal; Invitrogen). Relative quantification was done using the comparative 2^**-∆∆Ct**^ method [19] and expressed as fold change. The internal normalizer gene was 18S rRNA amplified with 18S primer forward (h18SRNA.891 F: GTTGGTTTTCGGAACTGAGG) and 18S reverse primer (h18SRNA.1090R: GCATCGTTTATGGTCGGAAC); amplification of SERCA2A/B/C was with primers forward (serca2abc7608F: CCTGTGCATGACTGATGTTG) and reverse (serca2abc7808R: CAGAGCCTCATTCCTCTTGC).

### IP_3_ mass assay

ASM cell monolayers in 24 well plates were stimulated with different concentrations of bradykinin (0.001-10 μM). Preliminary experiments demonstrated that IP_3_ accumulated rapidly after bradykinin addition and was maximal at 10 sec. Incubations were terminated by rapid aspiration and addition of trichloroacetic acid (0.5 M). Acid extracts were neutralized and IP_3_ accumulation measured using a competitive binding assay as described previously [20]. IP_3_ accumulation is expressed as pmol mg^−1^ of cell protein.

### Flash photolysis/Ca^2+^ uncaging

ASM cells plated at sub-confluence on coverslips were co-loaded with 2 μM Fluo-4 AM and 2.5 μM nitro-phenyl-EGTA AM (NP-EGTA AM; Invitrogen [18]) in the presence of 2.5 mM probenecid and 0.04% w/v pluronic F127 in physiological saline solution (see above for composition). Ca^2+^ transients were monitored using an Olympus confocal laser scanning inverted microscope (FV1000). The single scan head was used to monitor changes in [Ca^2+^]_i_ by detecting fluo-4 fluorescence with the 488 nm line of a multi-line argon laser (at ~2% maximum output) using ‘round-trip’ mode, with emission occurring at 528 nm and a wide confocal aperture setting of 374 μm. Near instantaneous switching allows photo-activation/uncaging, with a 405 nm laser flash without bleaching or response saturation, then switching back to 488 nm monitoring to capture the subsequent recovery of the cell towards basal [Ca^2+^]_i_ [17,21]. Intensity-time traces, with an image time interval of 32.77 ms, extending over 26 s were acquired with a × 60/1.2 NA oil-immersion objective with × 6 optical zoom. Uncaging pulses of the same intensity were delivered with the 405 nm laser (100%) for 300 ms in ‘tornado’ mode in a region of interest of diameter 15 pixels, selected in an area of the cytoplasm away from the nucleus. Each cell tested was only flashed once. On the microscope stage, coverslips were placed into a 1 mL chamber of an open perfusion microincubator (PDMI-2, Harvard Apparatus). Cells were maintained at 37°C and perfused at a rate of 3.6 mL min^−1^; where indicated cyclopiazonic acid (CPA, 10 μM, Sigma-Aldrich) was added to the perfusion solution. Intensity-time curves were analysed by performing a non-linear two phase exponential curve fit with a recovery rate K (s^−1^) derived for each trace. Mean K values were calculated for each donor/subject and comparison made between asthma and health/normal. Control experiments were performed using cells loaded with Fluo-4 AM only (no NP-EGTA AM) to confirm that the 405 nm flash did not perturb [Ca^2+^]_i_*per se*.

### Assessment of ASM contraction by collagen gel analysis

Collagen gels (299 μl of collagen (Inamed Biomaterials), 37 μl of 10X DMEM (Invitrogen), 20 μl of sodium bicarbonate (Invitrogen) were impregnated with 0.25 × 10^6^ ASM cells resuspended in 144 μl of serum-free medium with stimulus as required. The gels were added to 24-well plates (PBS/0.5% BSA) and left to polymerize at 37°C for 90 min. The gels were then detached and suspended in 500 μl of serum-free medium with stimulus as required. Bradykinin (Sigma-Aldrich) was added to appropriate wells to a final concentration of 1nM, with photographs taken at regular intervals up to 1 hour. The surface area of each gel was measured at each time point using ImageJ (http://rsb.info.nih.gov/ij) by a blinded observer.

### Statistical analysis

GraphPad Prism (version 6, GraphPad Software, San Diego) and IBM SPSS version 20 (SPSS, Inc., Chicago) were used to perform statistical analysis. Mean (standard error of the mean [S.E.M.]) was used to present parametric data, while median (interquartile ranges [IQR]) was used for non-parametric data. Comparisons between disease states used unpaired Student’s *t*-tests for parametric data, or Mann–Whitney tests for non-parametric data. Correlations were performed using either Pearson or Spearman of parametric and non-parametric data, respectively. A *P* value less than 0.05 was considered statistically significant.

## Results

**Figure 1:**
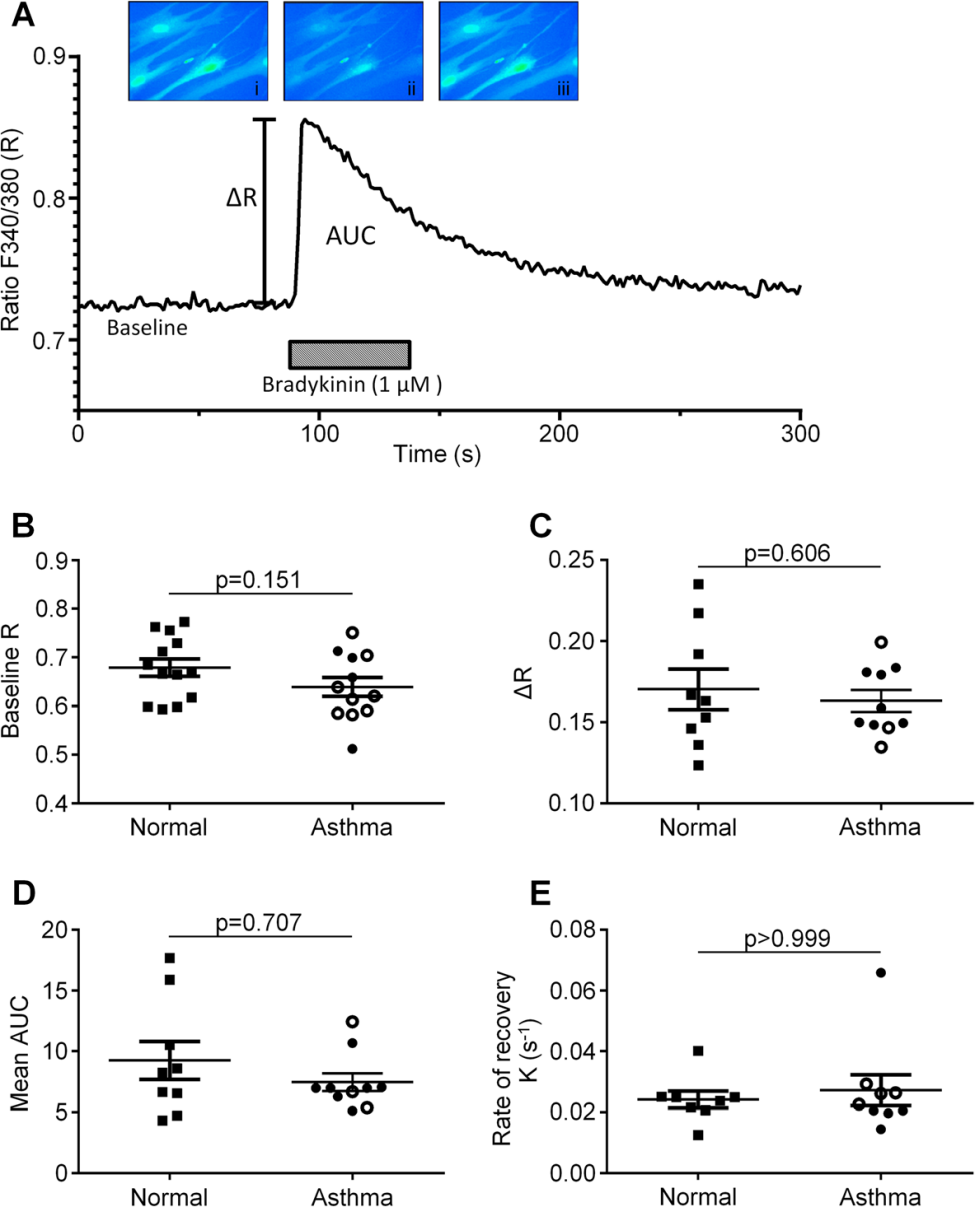
**Responses of ASM cells isolated from asthmatic and non-asthmatic subjects to bradykinin addition. (A)** Representative graph showing a [Ca^2+^]_i_ response to bradykinin (1 μM). Measurements taken from these Ca^2+^ responses were mean change in F_340 nm_/F_380 nm_ ratio (∆R), baseline Ca^2+^ concentration (expressed as F_340 nm_/F_380 nm_ ratio), area under the curve (AUC), and the rate of decline from the peak Ca^2+^ response. The baseline [Ca^2+^]_i_ **(B)**, peak increase in [Ca^2+^]_i_ following bradykinin stimulation **(C)**, AUC during the agonist addition **(D)**, and rate of decline in [Ca^2+^]_i_ from the peak bradykinin-stimulated response **(E)** were measured for each ASM cell donor across a range of cells (6–40 cells per donor) and the average value for each variable plotted for non-asthmatic and asthmatic donors. Symbol key: filled circles: GINA 1–3; open circles: GINA 4–5; filled squares: non-asthmatic donors. Parametric data **(B, C)** displayed as mean (SEM) and analysed by Student’s *t*-test. Non-parametric data **(D, E)** displayed as median (IQR) and analyzed using Mann–Whitney.

The clinical characteristics of the ASM donors are as shown in Table 1. An example trace showing the time-course of the [Ca^2+^]_i_ response following bradykinin addition is shown in Figure 1A. The average baseline [Ca^2+^]_i_ was determined for each donor used (Figure 1B) where a minimum of 5 cells per donor were analysed. No differences in F_340_/F_380_ ratio were found between health and disease (0.68 ± 0.02 and 0.64 ± 0.02, respectively; p = 0.15; Figure 1B), or indeed between ASM cells obtained from subjects with mild/moderate or severe asthma (0.65 ± 0.05 and 0.64 ± 0.02, respectively; p = 0.83; Figure 1B). When baseline [Ca^2+^]_i_ levels were correlated with FEV_1_% predicted and FEV_1_/FVC%, no correlations were identified (r = −0.03, p = 0.92 and r = 0.11, p = 0.74, respectively). Following addition of bradykinin (1 μM), the change in [Ca^2+^]_i_ (ΔR) was not different between non-asthmatic and asthmatic donors (change in F_340_/F_380_ ratio: 0.17 ± 0.01 and 0.16 ± 0.01, respectively; p = 0.61; Figure 1C), or between mild/moderate and severe asthma (0.16 ± 0.01 and 0.16 ± 0.02, respectively; p = 0.80; Figure 1C). The results for agonist-stimulated changes in [Ca^2+^]_i_ did not correlate with FEV_1_% predicted and FEV_1_/FVC% (r = −0.21, p = 0.56 and r = −0.19, p = 0.60, respectively). The area under the curve (AUC) values measured following bradykinin stimulation also did not significantly differ between health and disease (8.24[5.63-13.20] and 6.97[6.05-7.96], respectively; p = 0.71; Figure 1D), or between mild/moderate and severe asthma (6.97[6.27-7.05] and 8.67[6.03-9.72], respectively; p = 0.87; Figure 1D). These data also did not correlate with FEV_1_% predicted and FEV_1_/FVC% (r = −0.06, p = 0.86 and r = −0.06, p = 0.86, respectively). Finally, the rate of recovery following administration of bradykinin did not differ between ASM cells from non-asthma and asthma subjects (0.02[0.02-0.02] and 0.02[0.02-0.03], respectively; p > 0.99; Figure 1E), or between mild/moderate and severe asthma (0.02[0.02-0.04] and 0.03[0.02-0.03], respectively; p = 0.17; Figure 1E).

**Table 1:** **Clinical and functional characteristics of subjects**

**Characteristic**	**Normal**	**Mild-moderate asthma GINA 1-3**	**Severe asthma GINA 4-5**
**Number**	18	9	11
**Age**	48 (4.2)	43 (5.6)	48 (3.9)
**Male/Female**	10/8	3/6	9/2
**Smoking status**			
**Never/Ex/Current**	12/2/4	7/1/1	5/2/4
**Pack years** ^**+**^	29.0 (12.2)	5 (0.0)	19.5 (8.4)
**FEV**_**1**_ **% predicted**	97.0 (2.9)	89.1 (7.5)	95.2 (4.3)
**FEV**_**1**_ **/FVC%**	79.8 (1.7)	71.9 (3.3)*	69.6 (2.1)*
**Treatments**			
**LABA (% of patients)**	0	11	100
**ICS (% of patients)**	0	67	100
**Dose (bdp)**	-	533.3 (84.3)	1636 (152.1)
**OCS (% of patients)**	0	0	36
**Dose (mg/day)**	-	-	8.8 (0.7)

Data are mean (SEM), *p < 0.05 compared to normal group. ^+^Pack years refer to those subjects with a smoking history. FEV_1_, forced expiratory volume in one second; FVC, forced vital capacity; LABA, long-acting β_2_ adrenoceptor agonist; ICS, inhaled corticosteroid; OCS, oral corticosteroid.

**Figure 2:**
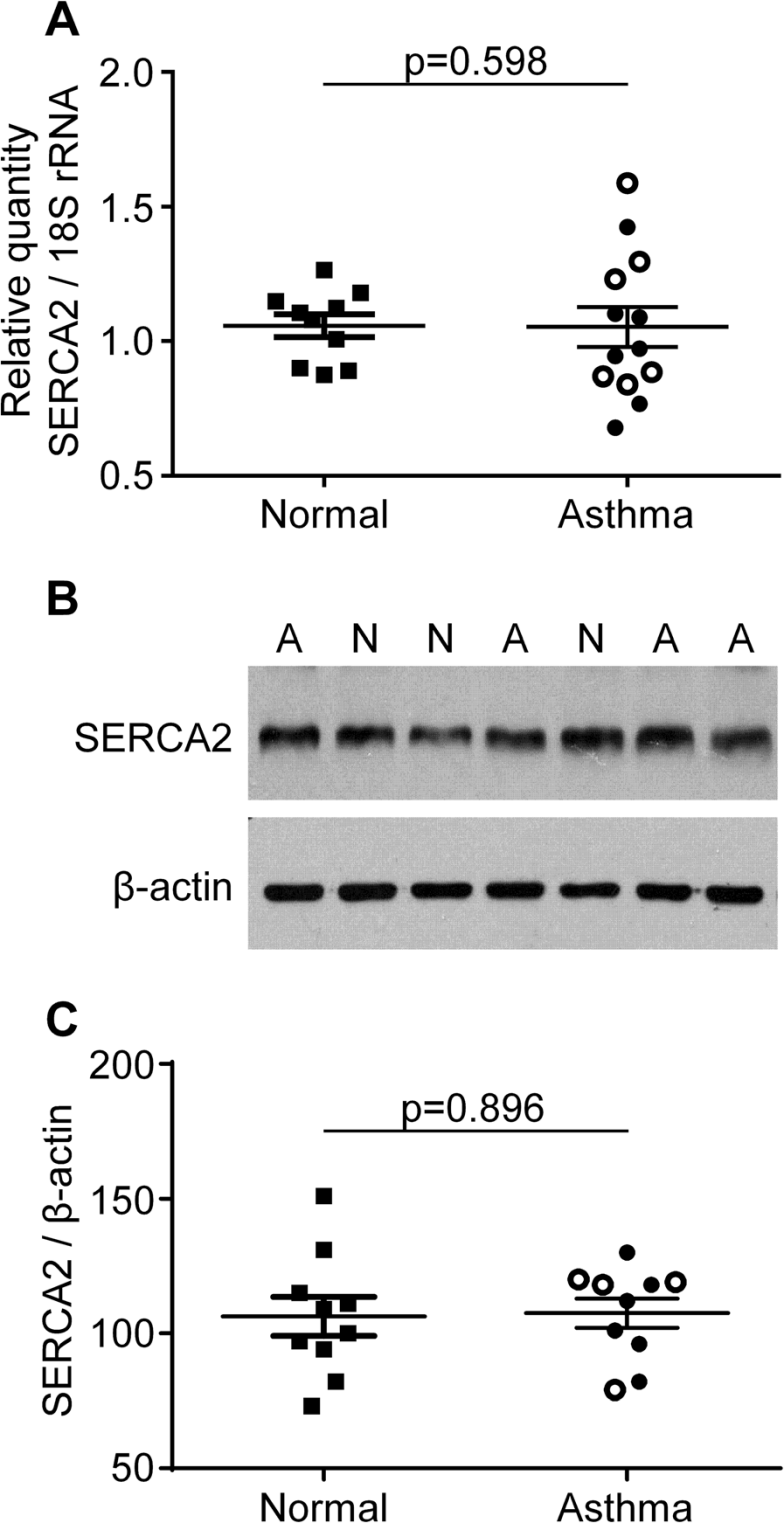
**Quantification of mRNA abundance and protein expression of SERCA2 in ASM cells from asthmatic and non-asthmatic subjects. (A)** The relative expression of SERCA2a/b/c mRNA normalized to 18S rRNA was not significantly different (Student’s *t*-test) in asthma (n = 13) compared to normal (n = 10) ASM donors. **(B)** An example western blot showing total SERCA2 protein and β-actin immunoreactivities in ASM cells from four asthma **(A)** and three normal (N) donors. **(C)** Densitometry of total SERCA2 immunoreactivity from western blots shows that there is no significant difference (Student’s *t*-test) in SERCA2 protein expression (normalized to β-actin expression) in asthma (n = 10) compared to normal (n = 10) ASM cell donors. Symbol key: filled circles: GINA 1–3; open circles: GINA 4–5; filled squares: non-asthmatic donors. Non-parametric data **(A)** displayed as median (IQR) and analyzed using Mann–Whitney. Parametric data **(C)** displayed as mean (SEM) and analyzed by Student’s *t*-test.

The relative expression of SERCA2A/B/C mRNA was assessed in 10 non-asthmatic control subjects and 13 patients with asthma (Figure 2A). Expression was not found to alter between health and disease (1.09[0.90-1.16] and 0.97[0.85-1.26], respectively; p = 0.60; Figure 2A), or between mild/moderate and severe asthma (0.97[0.77-1.10] and 1.06[0.86-1.37], respectively; p = 0.62; Figure 2A). There was no correlation either with FEV_1_% predicted and FEV_1_/FVC% (r = 0.37, p = 0.22 and r = −0.29, p = 0.32, respectively). Example western blots showing total SERCA2 protein and β-actin in ASM cells derived from four asthma patients and three non-asthmatic donors are shown in Figure 2B. Densitometry of total SERCA2 immunoreactivity from western blots showed that there was no significant difference in SERCA2 protein expression in normal (n = 10) compared to asthma (n = 10) ASM cell donors (106 ± 7 and 107 ± 5, respectively; p = 0.90; Figure 2C). Differences in protein expression were not found between asthma GINA 1–3 and GINA 4–5 (107 ± 7 and 109 ± 10, respectively; p = 0.84; Figure 2C). In addition, these data do not correlate with FEV_1_% predicted and FEV_1_/FVC% (r = −0.07, p = 0.85 and r = −0.01, p = 0.99, respectively).

**Figure 3:**
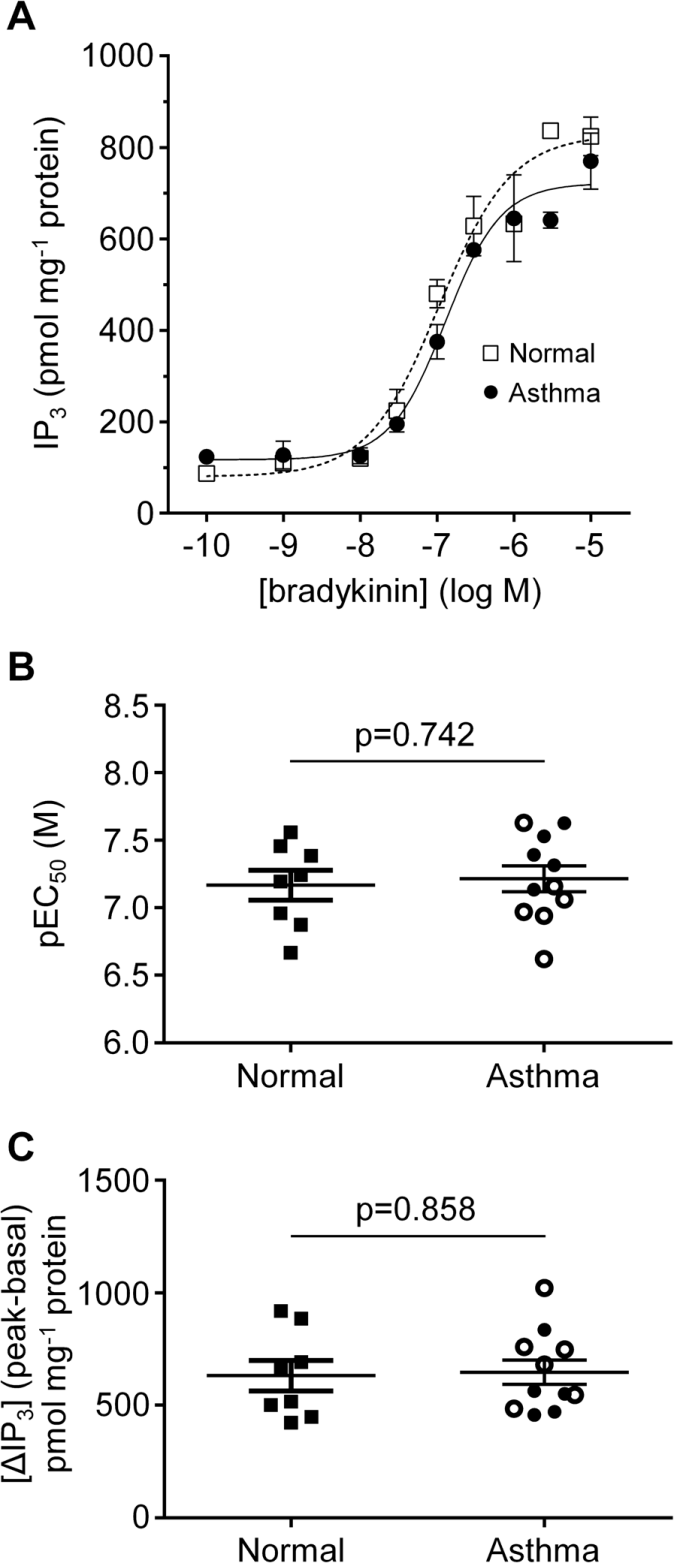
**Concentration-dependent IP**_**3**_ **accumulation responses to bradykinin in ASM cells derived from asthmatic and non-asthmatic donors. (A)** Representative examples of bradykinin concentration-response curves obtained for ASM cell monolayers from one asthmatic (○) and one non-asthmatic (▼) donor. **(B)** Graph showing the bradykinin concentrations causing a 50% of the maximal IP_3_ accumulation (shown as –log EC_50_ (M) values) in normal (n = 8) and asthmatic (n = 11) ASM donors. **(C)** The increase in IP_3_ accumulation following stimulation by a maximally effective concentration of bradykinin (10 μM) for ASM cells from non-asthmatic (n = 8) and asthmatic (n = 11) donors. Symbols key: filled circles: GINA 1–3; open circles: GINA 4–5; filled squares: non-asthmatic donors. Data are shown as mean (SEM) and analyzed by Student’s *t*-test.

Concentration-dependent IP_3_ accumulation responses to bradykinin were assessed in ASM cells derived from 8 non-asthmatic and 11 asthmatic patients. Typical concentration-response curves are shown for ASM cells obtained from a non-asthmatic or an asthmatic patient (Figure 3A). The bradykinin concentrations causing a half-maximal increase in IP_3_ accumulation (EC_50_) were not significantly different between non-asthmatic and asthmatic smooth muscle donors (−log EC_50_ (M): 7.17 ± 0.11 and 7.22 ± 0.10, respectively; p = 0.74; Figure 3B), nor between mild/moderate and severe asthma (7.40 ± 0.09 and 7.06 ± 0.14, respectively; p = 0.08). No correlation was found with FEV_1_% predicted and FEV_1_/FVC% (r = −0.38, p = 0.15 and r = −0.48, p = 0.14, respectively). The increase in IP_3_ accumulation (basal-to-peak; ∆IP_3_) following stimulation was not significantly different between health and disease (∆IP_3_ (pmol mg^−1^ protein): 631 ± 68 and 647 ± 54, respectively; p = 0.86; Figure 3C). Accumulation was not found to differ either between asthma GINA 1–3 and GINA 4–5 (576 ± 68 and 707 ± 77, respectively; p = 0.25). Maximal changes in IP_3_ accumulation stimulated by bradykinin did not correlate with FEV_1_% predicted and FEV_1_/FVC% (r = 0.01, p = 0.98 and r = −0.02, p = 0.96, respectively).

**Figure 4:**
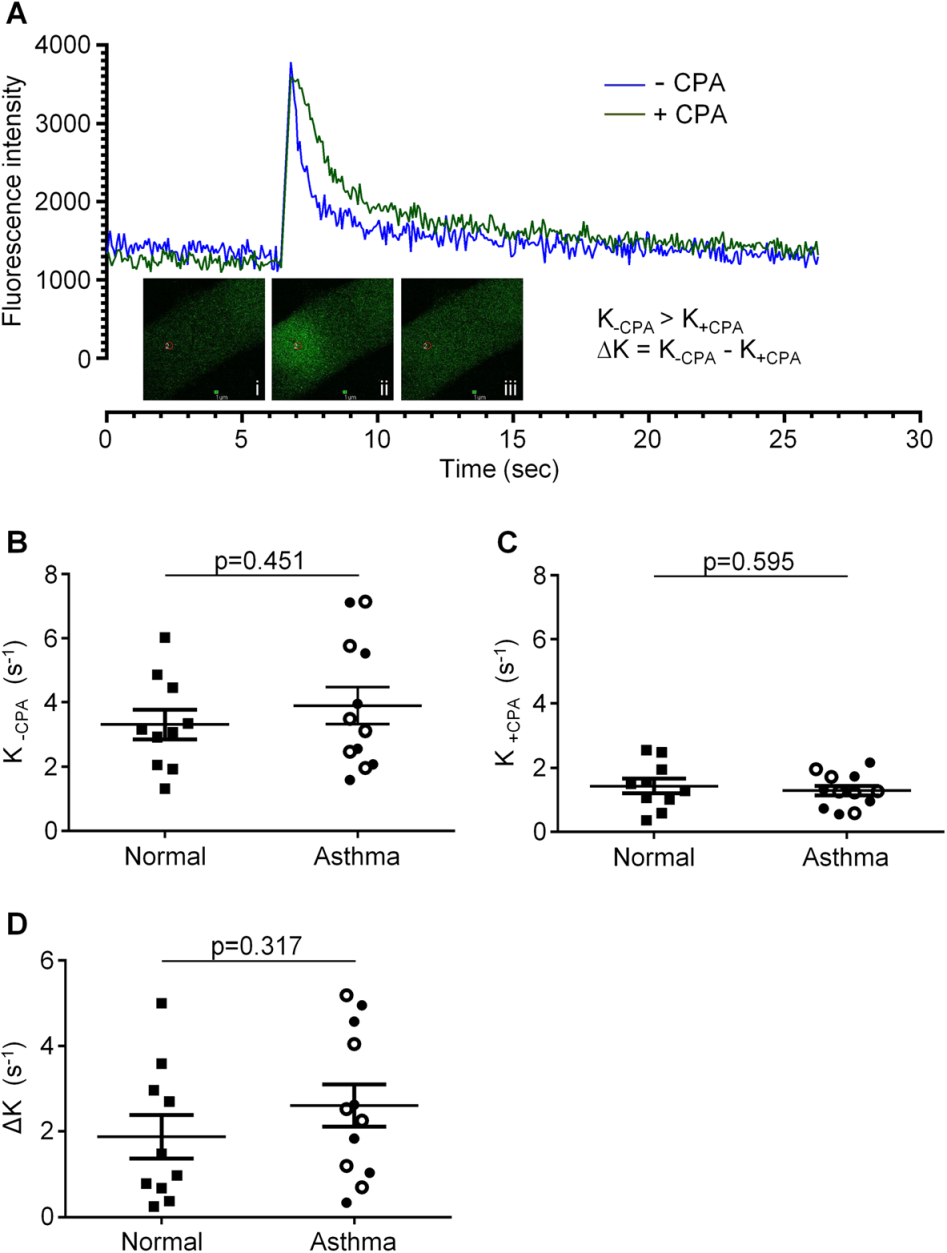
**Assessing the recovery of [Ca** ^**2+**^ **]**_**i**_ **following Ca** ^**2+**^ **-uncaging in ASM cells derived from asthmatic and non-asthmatic donors. (A)** Representative examples of changes in [Ca^2+^]_i_ on photolysis of the NP-EGTA Ca^2+^ cage and the subsequent rate of recovery to pre-uncaging levels of [Ca^2+^]_i_. Changes in [Ca^2+^]_i_ are shown for ASM cells in the absence (blue line) or presence (green line) of the SERCA inhibitor, CPA (10 μM). Inset example photographs of Ca^2+^ uncaging using a confocal microscope before (i), during (ii) and after (iii) the 405 nm laser flash. **(B)** Mean Ca^2+^ uncaging recovery rates (K_-CPA_) without SERCA inhibition in ASM cells from normal (n = 10) and asthma (n = 10) donors. **(C)** Mean Ca^2+^ uncaging recovery rates when SERCA is inhibited (K_+CPA_) in ASM cells from normal (n = 10) and asthma (n = 12) donors. **(D)** The difference between K_-CPA_ and K_+CPA_ (∆K), which represents primarily trans-plasmalemmal efflux of Ca^2+^ in ASM cells, is also plotted for each normal (n = 10) and asthmatic (n = 12) donor. Symbol key: filled circles: GINA 1–3; open circles: GINA 4–5 filled squares: non-asthmatic donors. Data are shown as mean (SEM) and analyzed by Student’s *t*-test.

Comparisons of [Ca^2+^]_i_ recovery rates following Ca^2+^ uncaging in the absence or presence of SERCA inhibition were made in ASM cells from patients with asthma or non-asthmatic controls (example traces shown in Figure 4A). There was no significant difference in the mean Ca^2+^ uncaging recovery rate between health and disease either in the absence of CPA (K_-CPA_ (s^−1^): 3.31 ± 0.46 and 3.89 ± 0.58, respectively; p = 0.45; Figure 4B), or when SERCA was inhibited, (K_+CPA_ (s^−1^): 1.43 ± 0.23 and 1.29 ± 0.15, respectively; p = 0.60; Figure 4C). Similarly, no differences were seen in recovery rates between mild/moderate and severe asthma in the absence (K_-CPA_: 3.80 ± 0.88 and 3.99 ± 0.83, respectively; p = 0.88) or presence (K_+CPA_: 1.24 ± 0.25 and 1.34 ± 0.19, respectively; p = 0.77; Figure 4C) of SERCA inhibition. The difference between K_-CPA_ and K_+CPA_ (∆K) values in ASM cells from non-asthmatic and asthmatic patients did not reveal any differences (∆K: 1.88 ± 0.51 and 2.61 ± 0.49, respectively; p = 0.32), nor was there a difference between asthma GINA classification 1–3 and 4–5 (∆K: 2.56 ± 0.76 and 2.65 ± 0.69, respectively; p = 0.93).

**Figure 5:**
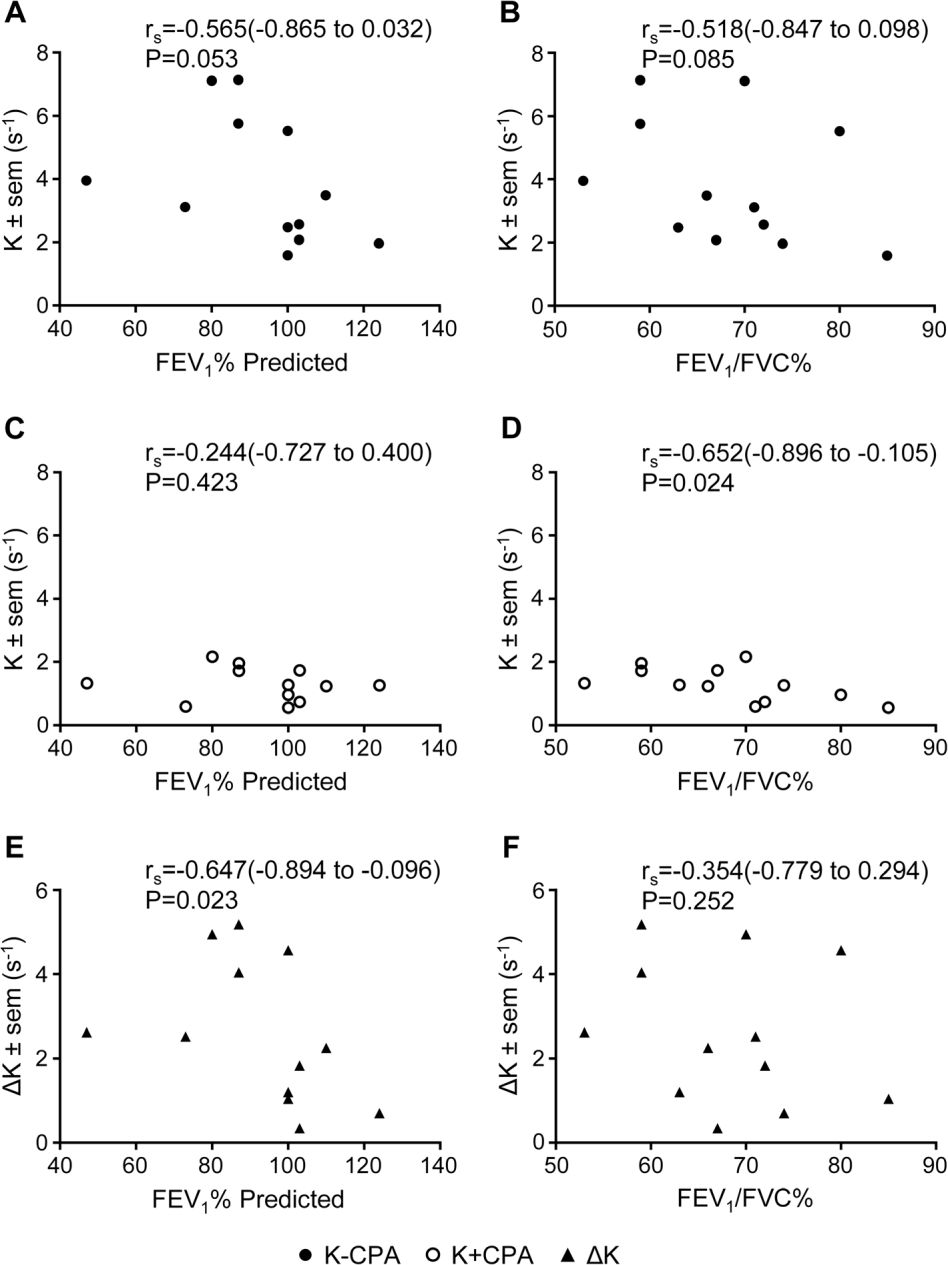
**Correlations of rates of recovery following Ca** ^**2+**^ **uncaging in ASM cells with clinical lung function parameters in respective patient donors.** Recovery rates compared to the FEV_1_% predicted **(A, C, E)** and FEV_1_/FVC% **(B, D, F)** are shown in the absence of inhibitor (−CPA, ●, **A**, **B**), in the presence of CPA (+CPA, ○, **C**, **D**), or for the difference between –CPA and + CPA recovery rates (ΔK, ▲, **E, F**). Data were analyzed by Spearman correlation (95% confidence interval).

Correlations were observed in the subjects with asthma between lung function and Ca^2+^ uncaging recovery rates with and without SERCA inhibition by CPA (Figure 5). There was a trend correlating the mean Ca^2+^ uncaging recovery rate with FEV_1_% predicted (r_s_ = −0.565, p = 0.053; Figure 5A), which was no longer observed when SERCA activity was inhibited (r_s_ = −0.244, p = 0.423; Figure 5C). A trend was also observed between the FEV_1_/FVC% and Ca^2+^ uncaging recovery rates (r_s_ = −0.518, p = 0.085; Figure 5B), which was statistically significantly correlated when SERCA activity was inhibited (r_s_ = −0.652, p = 0.024; Figure 5D). When the difference between the mean Ca^2+^ uncaging recovery rates with and without CPA (∆K) was compared with FEV_1_% predicted, there was again a significant negative correlation (r_s_ = −0.647, p = 0.023; Figure 5E), but not with FEV_1_/FVC% (r_s_ = −0.354, p = 0.252; Figure 5F).

**Figure 6:**
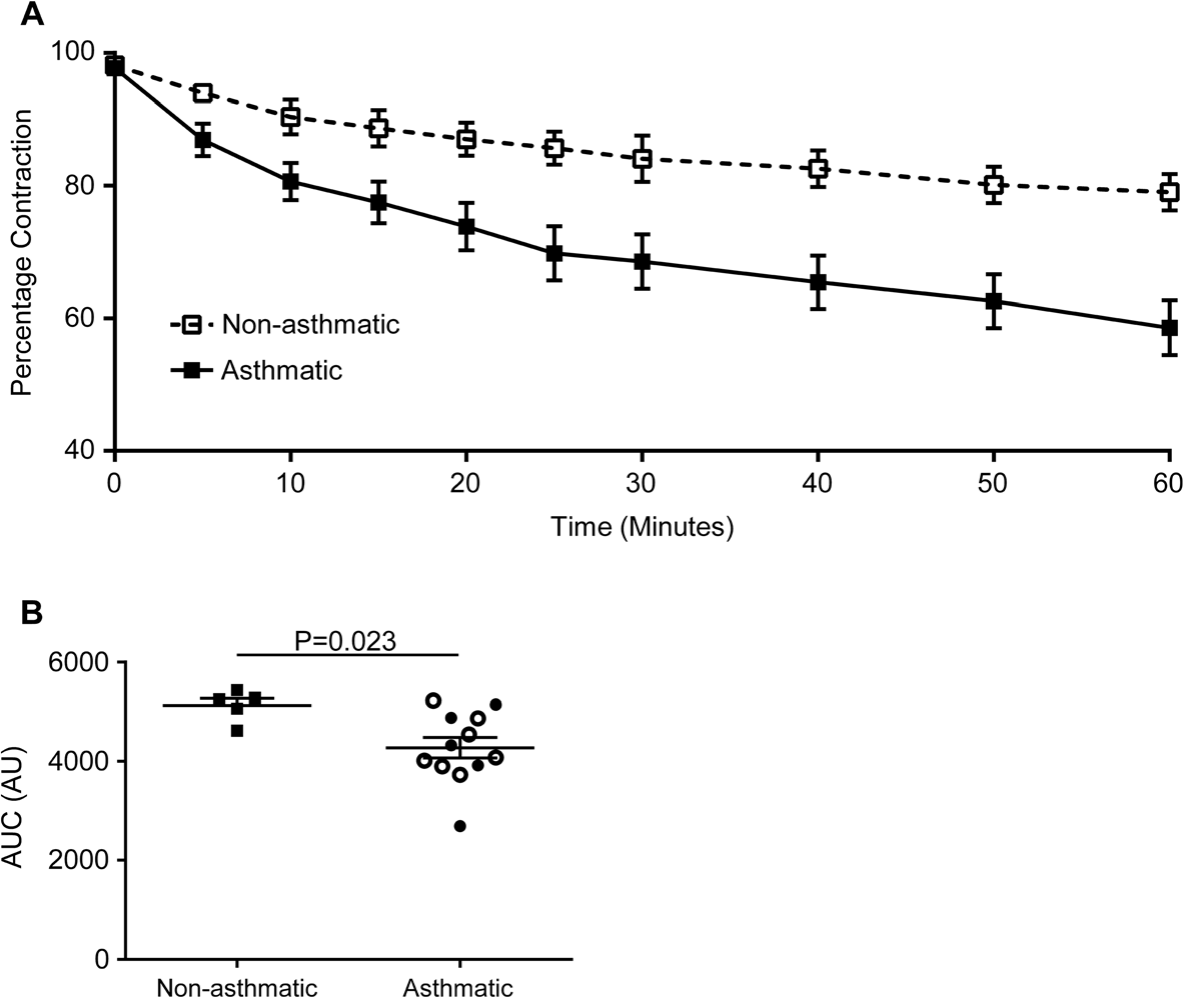
**Collagen gel contraction assay of human airway smooth muscle cells. (A)** Percentage contraction of collagen gels impregnated with airway smooth muscle from donors who are not asthmatic (n = 5) and donors with asthma (n = 12) over 1 hour following stimulation with 1 nM bradykinin. **(B)** Area under the curve gel contraction. Symbol key: filled circles: GINA 1–3; open circles: GINA 4–5; filled squares: non-asthmatic donors. Data are shown as mean ± SEM and analysed by Student’s *t*-test.

In 17/38 ASM donors, agonist-induced contraction was assessed. The ASM from asthmatic was hypercontractile (mean difference in area under the curve contraction ± SEM; 5127 ± 140; 4274 ± 207; p = 0.023; non-asthmatic compared to asthmatic respectively; Figure 6A and B). Area under the curve contraction was not related to ∆R, but was related to K and ∆K (r = 0.04; p = 0.93; r_s_ = −0.691; p = 0.023; r_s_ = −0.618; p = 0.048 respectively; data not shown).

## Discussion

Here we have studied [Ca^2+^]_i_ handling by low passage primary ASM cells from subjects with asthma compared to non-asthmatic controls. Our findings demonstrate no differences in basal [Ca^2+^]_i_, agonist-induced peak [Ca^2+^]_i_ responses or recovery following agonist activation. Concentration-dependent accumulations of the second messenger IP_3_ in response to bradykinin addition were not different in ASM cells from subjects with asthma versus non-asthmatic controls, although there was a small, significant increase in maximal bradykinin-stimulated IP_3_ responses when severe (GINA 4–5) and mild-moderate (GINA 1–3) asthma patients were compared. We did not identify any differences in SERCA2 mRNA or protein expression. [Ca^2+^]_i_ kinetics assessed by flash photolysis and calcium uncaging independent of agonist-activation again did not demonstrate differences in rates of recovery of [Ca^2+^]_i_ either with or without SERCA inhibition. However, we did observe correlations in the subjects with asthma between lung function and [Ca^2+^]_i_ rate of recovery following flash photolysis with poorer lung function relating to an increased rate of recovery. Taken together this study does not support a fundamental difference in [Ca^2+^]_i_ handling in ASM from subjects with asthma compared to non-asthmatic controls *per se*, but does not exclude a possible contribution in specific phenotypes, such as those with impaired lung function, as suggested here by subtle differences in [Ca^2+^]_i_ recovery rates.

Our findings of no disease-related differences in agonist-induced evoked changes in [Ca^2+^]_i_ conflicts with work published previously [11,15,16]. Mahn and colleagues reported a reduced peak [Ca^2+^]_i_ response to the same concentration of bradykinin [11], a reduced response to ryanodine (1 μM) and thapsigargin (0.1 μM) in ASM cells from asthmatic versus non-asthmatic donors. Similarly, Trian *et al.* reported a reduced [Ca^2+^]_i_ response to acetylcholine (10 μM) or histamine (10 μM) in ASM cells from asthmatic subjects [16]. Interestingly, in contrast to our report, the healthy control and asthma subjects were not age matched and whether calcium handling is altered by age of the donor is unknown. We have been unable to confirm these earlier findings and did not observe associations between agonist-induced [Ca^2+^]_i_ peak or rate of recovery with severity of disease defined by GINA treatment steps, nor with lung function. Our study had 80% power to observe a 0.05 difference between health and disease in [Ca^2+^]_i_ (ΔR) following bradykinin activation. Mahn *et al.* reported [Ca^2+^]_i_ (ΔR) of 0.07 and 0.12 between ASM from non-asthmaticsubjects versus those with either mild or moderate asthma, respectively [11]. Therefore, our study had sufficient power to observe the differences reported previously. Thus, it would seem likely that the differences found here compared to earlier reports reflect the heterogeneity of the disease and indicate that changes in [Ca^2+^]_i_ in response to agonist might be altered in some asthmatics. Therefore, whether altered [Ca^2+^]_i_ handling is typically altered in asthma requires further study in a larger population.

In addition to our inability to replicate earlier reports of differences in agonist-evoked changes in [Ca^2+^]_i_ we were also unable to demonstrate differences in SERCA2 expression, either at the mRNA or protein levels. Mahn *et al.* found reduced expression of both mRNA and protein in primary ASM cells and reduced expression in ASM *in vivo* in tissue sections of endobronchial biopsies, in both moderate and severe asthmatics compared to non-asthmatic controls [11]. We did not investigate SERCA2 expression in tissue and therefore cannot exclude the possibility of differential expression *in vivo*, but our findings do not support a fundamental change in SERCA expression in disease, nor a relationship with disease severity determined by either treatment requirement or lung function. Again our study was sufficiently powered to observe the differences described by Mahn *et al.* and therefore, the most plausible explanation is that decreased SERCA expression is not a consistent feature of ASM from subjects with asthma.

We report for the first time a comparison of the concentration dependency and magnitude of changes in the Ca^2+^-mobilizing second messenger IP_3_ in response to bradykinin in ASM cells from subjects with asthma compared to health. We did not find any differences between health and disease, or disease severity. Here we also extended the exploration of intracellular [Ca^2+^]_i_ handling to investigate the differences in ASM cells from subjects with asthma and non-asthmatic controls independent of agonist. [Ca^2+^]_i_ kinetics assessed by flash photolysis and calcium uncaging independent of agonist-activation again did not demonstrate differences in rates of recovery of [Ca^2+^]_i_ either with or without SERCA inhibition. Interestingly, we did observe correlations in the subjects with asthma between lung function and rates of recovery of [Ca^2+^]_i_ following flash photolysis. Those asthmatics with poorer lung function had greater rates of [Ca^2+^]_i_ recovery. This again conflicts with the report from Mahn *et al.*, which suggests that rates of recovery are reduced in asthma [11,15]. Similar correlations between spontaneous calcium oscillation frequency and lung function were observed previously [22]. Whether there is a common mechanism between spontaneous oscillations and rate of recovery in [Ca^2+^]_i_ following flash photolysis requires further investigation. However, although the correlations reported here are intriguing, they are only observed in those ASM cells from subjects with asthma and there were no differences in uncaging [Ca^2+^]_i_ rate of recovery between asthma and health, questioning whether these relationships are important.

In contrast to the inconsistency of the observations relating to [Ca^2+^]_i_ handling in ASM cells from subjects with asthma, we and others have consistently found that primary ASM cells from asthmatics are hyper-contractile [5,9-11]. Contraction in ASM is driven by [Ca^2+^]_i_ originating from the sarcoplasmic reticulum stores, therefore, the hyper-contractility seen in asthma is likely to be due to abnormalities in [Ca^2+^]_i_ handling, Ca^2+^ sensitivity or changes to the contractile machinery. Since we have not shown here any intrinsic abnormality in [Ca^2+^]_i_ or SERCA2 in ASM cells derived from asthmatic patients when compared with non-asthmatic subjects, we must consider alternative mechanisms. Myosin light-chain kinase (MLCK) is a major player in this Ca^2+^/contraction pathway and indeed others have found expression of MLCK to be increased [11,23]. Up-regulation of the Ca^2+^-independent rhoA/rho kinase signalling pathway leads to inhibition of myosin light-chain phosphatase resulting in increased levels of phosphorylated myosin light-chains and increased ASM contraction force at a given increase in [Ca^2+^]_i_ [24]. Abnormalities within this pathway have been reported in animal models [25,26]. Beyond the Ca^2+^/contraction pathway, increased production of reactive oxygen species (ROS) can damage tissue; indeed, the oxidative burden is increased in asthma. Nicotinamide adenine dinucleotide phosphate oxidase 4 (NOX4), an important source of ROS, is increased in asthmatics and hyper-contractility of ASM from asthmatic patients, seen in gel-contraction assays, could be abolished following the addition of NOX4 inhibitors or transfection with small interfering RNAs targeting NOX4 [5]. Pathways, such as cAMP signalling that mediate bronchorelaxation, for example cAMP dependent PKA inhibits MLCK by reducing its affinity for Ca^2+^/CaM (reviewed in [27]), may also be differentially regulated in ASM from subjects with asthma versus health. Furthermore, phosphodiesterase (PDE)4 and prostaglandin (PG)E_2_ are differentially expressed in ASM from asthmatic and healthy donors, and might contribute to increased contration in asthma [28,29]. These and other mechanisms need to be explored further in future studies.

Our study has a number of potential limitations. We have used primary ASM cells and therefore whether important mechanisms present *in vivo* persist in culture is uncertain. However, our ability to observe hyper-contractility in primary ASM from asthmatics does suggest the mechanism driving this abnormality persists *in vitro* and therefore our inability to demonstrate altered [Ca^2+^]_i_ handling remains important. Perhaps more critical is the heterogeneity of asthma and even though our study has sufficient power to observe large differences in [Ca^2+^]_i_ handling we might have failed to observe small, albeit important, differences in specific phenotypes of asthma. For example, our intriguing observations of correlations between lung function and [Ca^2+^]_i_ rates of recovery following NP-EGTA Ca^2+^ uncaging might reflect important associations in a sub-group of asthmatics.

## Conclusions

In summary, we have not shown major fundamental differences in Ca^2+^ handling between ASM from subjects with and without asthma, in contrast to previous reports [11,16], we conclude that differences in contraction between asthmatic and non-asthmatic subject-derived primary ASM cells cannot be fully explained by altered [Ca^2+^]_i_ homeostasis in asthma.

## Abbreviations

[Ca^2+^]_i_: Cytosolic calcium concentration
ASM: Airway smooth muscle
AUC: Area under the curve
cAMP: Cyclic adenosine monophosphate
CPA: Cyclopiazonic acid
F: Fluorescence
FEV_1_: Forced expiratory volume in one second
FVC: Forced expiratory vital capacity
GINA: Global Initiative for Asthma
IP_3_: inositol 1,4,5-trisphosphate
MLCK: Myosin light-chain kinase
NOX4: Nicotinamide adenine dinucleotide phosphate oxidase 4
PKA: Protein kinase A
R: Ratio
ROS: Reactive oxygen species
SERCA: sarco/endoplasmic reticulum Ca^2+^-ATPase
